# Effects of microcurrent stimulation on Hyaline cartilage repair in immature male rats (*Rattus norvegicus*)

**DOI:** 10.1186/1472-6882-13-17

**Published:** 2013-01-19

**Authors:** Carla de Campos Ciccone, Denise Cristina Zuzzi, Lia Mara Grosso Neves, Josué Sampaio Mendonça, Paulo Pinto Joazeiro, Marcelo Augusto Marretto Esquisatto

**Affiliations:** 1 Programa de Pós-graduação em Ciências Biomédicas, Centro Universitário Hermínio Ometto, Av. Dr. Maximiliano Baruto, 500 Jd. Universitário, 13607-339, Araras, SP, Brazil; 2 Departamento de Histologia e Embriologia, Instituto de Biologia, Universidade Estadual de Campinas, Rua Charles Darwin, s/n. CxP 6109, 13083-863, Campinas, SP, Brazil

**Keywords:** Hyaline cartilage, Tissue repair, Extracellular matrix, Electrotherapy, Immature rats

## Abstract

**Background:**

In this study, we investigate the effects of microcurrent stimulation on the repair process of xiphoid cartilage in 45-days-old rats.

**Methods:**

Twenty male rats were divided into a control group and a treated group. A 3-mm defect was then created with a punch in anesthetized animals. In the treated group, animals were submitted to daily applications of a biphasic square pulse microgalvanic continuous electrical current during 5 min. In each application, it was used a frequency of 0.3 Hz and intensity of 20 μA. The animals were sacrificed at 7, 21 and 35 days after injury for structural analysis.

**Results:**

Basophilia increased gradually in control animals during the experimental period. In treated animals, newly formed cartilage was observed on days 21 and 35. No statistically significant differences in birefringent collagen fibers were seen between groups at any of the time points. Treated animals presented a statistically larger number of chondroblasts. Calcification points were observed in treated animals on day 35. Ultrastructural analysis revealed differences in cell and matrix characteristics between the two groups. Chondrocyte-like cells were seen in control animals only after 35 days, whereas they were present in treated animals as early as by day 21. The number of cuprolinic blue-stained proteoglycans was statistically higher in treated animals on days 21 and 35.

**Conclusion:**

We conclude that microcurrent stimulation accelerates the cartilage repair in non-articular site from prepuberal animals.

## Background

Most studies on cartilage repair use models of osteochondral defects since these defects are a major public health problem. Although these models permit to monitor the integration of articular cartilage under conditions of compressive load, they do not provide information about the characteristics of the newly formed tissue and its integration into the surrounding preserved tissue [1].

Hyaline cartilage is a highly specialized, aneural and avascular connective tissue derived from the embryonic mesenchyme. Morphologically, this tissue is characterized by the presence of a small number of cells, called chondrocytes, which are responsible for the production, organization and renewal of extracellular matrix (ECM). The latter corresponds to most of the tissue’s dry weight, surrounding all chondrocytes and maintaining a strong structural and functional relationship with these cells [2,3].

The ECM is formed by a complex of macromolecules including collagens, proteoglycans (PGs) and non-collagen proteins. The interaction of these components ensures that an adequate amount of water is retained inside the matrix and is responsible for the functional properties of the tissue [2,4].

Chondrocytes are nourished by the diffusion of nutrients through the ECM, either from synovial fluid or from capillaries in the perichondrium [5,6].

The cartilaginous ECM consists of fibrillar elements such as collagen and elastin and PGs. The main types of collagen found in cartilage are, in decreasing order, types II, IX and XI. Collagen fibrils and fibers provide resistance to tensile loading and sustain the organization of the cartilaginous stroma [2,6]. The organization, composition and concentration of the main ECM components are intimately related to the biomechanical properties of the tissue [4,7-9]. Compressive loads applied to the surface of articular cartilage associated with repetitive and inadequate movements, as well as the natural process of aging, directly influence the organization of cartilage tissue [10-12]. Clinical evidence indicates that small-diameter cartilage defects induced in young animals tend to stimulate cartilage repair, which results in the formation of new tissue with similar properties. This finding was attributed to the proliferative capacity, mitotic division and ECM synthesis induced by the accelerated metabolism of chondrocytes [5,13,14]. However, the absence of blood supply limits the capacity of cartilage repair in older animals after different types of injuries [1,12,14,15]. Articular surface defects that do not penetrate the subchondral bone do not heal spontaneously. On the other hand, injuries that penetrate the subchondral bone exhibit an intrinsic repair potential because of an increased blood supply. However, the newly formed tissue consists of fibrocartilage and does not possess the same biochemical characteristics as native cartilage [1,12,16-18].

Non-articular cartilage differs from articular cartilage by the fact that it is does not suffer the wear and tear of weight-bearing articular cartilage and that the defects induced are chondral and not osteochondral [19]. Chondrocytes isolated from non-articular cartilage contain larger amounts of lipid and glycogen inclusions due to the slower metabolism of this tissue [20,21]. In addition, studies have shown differences in the structure of ECM molecules isolated from cartilage obtained from anatomical sites that withstand compressive loads and those that do not [22]. The extracellular presence of adenosine triphosphate (ATP) indicates recovery of articular cartilage ECM and, at the same time, degradation of cartilage of other origins [23].

There are various treatment options for articular cartilage defects. Electrical and electromagnetic stimulation and autologous grafts of chondrocytes, mesenchymal cells and biocompatible tissue derived from the periosteum and perichondrium show a marked chondrogenic potential [12,24]. However, in contrast to electrical stimulation, the implantation of cells and tissues of different origins requires invasive procedures [14,15]. Within this context, electrical stimulation as a therapeutic strategy for cartilage repair has been little investigated [6,25,26] and knowledge about the response of cartilage tissue of different anatomical origins to treatment with physical agents, particularly the application of electrical currents of different intensities and for different periods of time, is scarce [25,27]. Similarly, there are no reliable reports in the literature regarding the adequate parameters and type of electrical current that would promote the regeneration of articular and non-articular cartilage [25,28].

Therefore, the objective of the present study was to investigate the structural and ultrastructural alterations that occur during the healing of non-articular cartilage defects after microcurrent stimulation in prepubertal rats.

## Methods

### Experimental groups

Twenty male Wistar rats, 45 days old and weighing 150 to 200 g, were obtained from the Center of Animal Experimentation, Centro Universitário Hermínio Ometto, UNIARARAS. The animals were housed in individual cages and received commercial ration and water *ad libitum*. For the study, the animals were divided into two groups of 10 rats each: a control group and a treated group submitted to microcurrent stimulation. The protocol (n. 073/2010) was approved by the UNIARARAS Ethics Committee on Animal Use (ECAU/CONCEA-MCTI, Brazil).

### Experimental injury

The animals were anesthetized with xylazine hydrochloride (0.2 mg/kg) and ketamine hydrochloride (1 mg/kg). After the ventral region was shaved at the height of the sternum, a 2-cm incision was made in the lower area of the sternum in the parasagittal direction and the skin was folded back to expose the abdominal muscles and xiphoid process. The latter was separated carefully from the peritoneum. Surgery was performed maintaining the integrity of blood vessels. Finally, the xiphoid process was separated completely in such a way to preserve the perichondrium. The cartilage was exposed with the help of a spatula and a cylindrical defect (3 mm in diameter and 2 mm thick) was created at the caudal end of the xiphoid cartilage with a surgical punch. At the end of the procedure, the perichondrium was repositioned and the peritoneum, abdominal muscles and skin were sutured. For analgesia, the animals received 0.5% sodium dipyrone in their drinking water (1:1,000) for the first 3 days after surgery. All surgical instruments were sterilized to prevent contamination of the wounds.

### Electrical current stimulation

Animals of the treated group received daily applications of a biphasic square pulse microgalvanic continuous electrical current during 5 min (Physiotonus Microcurrent© stimulator - Bioset, Rio Claro, Sao Paulo, Brazil). In each application, it was used a frequency of 0.3 Hz and intensity of 20 μA. The pulse duration was the 10s with a interpulse interval of the 2 s. For this purpose, the animals were anesthetized using half the dose employed for the surgical procedure. During current application, two metal electrodes, with a spherical tip (10 mm), were placing on the left and right of the lesion for 2.5 min and then, the position was reversed and current was applied for a further 2.5 min. This apparatus generates a sub-sensory current that does not excite peripheral nerves. Treatment of the experimental animals was started 24 h after surgical intervention and lasted for 35 days [19]. The animals from control group were submitted in the same way as the treated group, but the electric current was not applied. All the animals were sacrificed with an overdose of the anesthetic at 7, 21 and 35 days after experimental injury, and the xiphoid cartilage was removed for structural and ultrastructural analysis.

### Macroscopic inspection

Xiphoid cartilage specimens fixed for histochemical analysis were used. The specimens were photographed with a PinePIX 300 digital camera in the front view. Anatomical features, including the appearance of the newly formed tissue compared to the original tissue, were analyzed.

### Histochemistry analysis

After removal, the xiphoid cartilage fragments were fixed in 10% formaldehyde in Millonig buffer, pH 7.4, for 24 h at room temperature. The specimens were then transferred to buffer and processed routinely for embedding in Paraplast® (Merck, Darmstadt, Germany). Longitudinal sections (6 μm) were cut and stained with the following dyes: picrosirius-hematoxylin for microscopic examination under polarized light and observation of collagen fiber organization; Toluidine blue in McIlvaine buffer, pH 4.0, for analysis of acid glycosaminoglycans (basophilia); Verhoeff stain for analysis of the elastic fiber system; Dominici stain for determination of the number of granulocytes, and Alizarin red for the detection of calcium deposits. The specimens were analyzed and documented with a Leica DM2000 photomicroscope at the Laboratory of Micromorphology, Centro Universitário Hermínio Ometto, UNIARARAS.

### Electron microscopy and cytochemistry study

The cartilage samples of the two groups were fixed in 2% glutaraldehyde and 0.1% tannic acid dissolved in 0.1 M sodium cacodylate buffer, pH 7.3, for 2 h at room temperature. Next, the material was washed in buffer and postfixed in 1% osmium tetroxide for 1 h at 4°C. After this step, the fragments were washed in glycated saline, treated with 1% uranyl acetate for 18 at 4°C, again washed in glycated saline, and dehydrated. Fragments of the same samples were submitted to cytochemical staining using cuprolinic blue for the ultrastructural detection of PGs [29]. After fixation and postfixation, the samples were dehydrated in a growing ethanol series and dual passage through propylene oxide. The specimens were embedded in mixtures of propylene oxide/Epon resin (1:1, 1:2, and pure) and transferred to plastic casts in an oven (60°C). Sections were cut with the Leica RM2245 and Ultracut UCT ultramicrotomes using glass and diamond knives and counterstained with 2% uranyl acetate in water and 0.2% lead citrate in 0.1 N NaOH. The sections were examined and the images were documented with a Leica electron transmission microscope (JEOL 4507) at the Center of Electron Microscopy, Institute of Biology, UNICAMP.

### Morphometric and statistical analysis

The cartilage repair area was determined on the digitized images as percent reduction of the original defect. Images of longitudinal sections stained with Toluidine blue, Dominici stain and picrosirius-hematoxylin were used to evaluate the number of fibroblasts cells, granulocytes (n/10^4^ μm^2^) and the area occupied by birefringent collagen fibers in the repair tissue (%), respectively, in the two groups. For each group, three samples were collected from each section obtained from the entire specimen of each animal. All images were captured and digitized with a Leica DM2000 photomicroscope. In addition, the diameter of collagen fibrils (nm) and the number of stained PGs (n/25 μm^2^) were measured on electron micrographs [29]. The measurements were made on digitized images using the Sigma Scan PRO 6.0™ program. The results were entered into spreadsheets of the Excel, Statistics Module, for Windows XP ™ program and analyzed using ANOVA and the Tukey post-test (*p* < 0.05). Data are reported as the mean and respective standard deviation.

## Results

Macroscopic inspection of the wound area showed the circular shape of the defect in control and treated animals and the absence of hemorrhagic or infectious processes at any of the time points studied (data not shown). No significant difference in defect area reduction was observed between groups or between time points (Table 1).

**Table 1:** Morphometric parameters evaluated in the defect area after different periods of time

**Parameter**	**Time**	**Control group**	**Treated group**
	7d	2.5 ± 1.1	2.9 ± 1.8
Lesion reduction (%)	21d	31.8 ± 10.6	36.4 ± 9.8
	35d	44.5 ± 11.8	49.8 ± 13.7
	7d	23.4 ± 6.9	33.2 ± 7.8* ^p=**0,048**^
Fibroblastic cells	21d	47.4 ± 7.3	60.6 ± 8.1* ^p=**0,041**^
(n/10^4^ μm^2^)	35d	91.5 ± 12.8	107.8 ± 10.7* ^p=**0,036**^
	7d	37.4 ± 5.9	84.2 ± 9.8* ^p=**0**,**021**^
Granulocytes	21d	38.4 ± 7.3	54.6 ± 8.1* ^p=**0,042**^
(n/10^4^ μm^2^)	35d	18.5 ± 3.8	19.8 ± 4.7
	7d	47.4 ± 11.9	44.2 ± 9.8
Birefringent collagen fibers	21d	48.4 ± 9.3	47.6 ± 8.1
(%)	35d	87.5 ± 7.8	69.3 ± 9.7* ^p=**0,045**^
	7d	39.4 ± 10.9	41.2 ± 12.1
Collagen fibril diameter	21d	40.4 ± 12.3	41.6 ± 12.6
(nm)	35d	40.9 ± 11.8	42.3 ± 13.7
	7d	3.4 ± 1.9	4.2 ± 1.1
Stained proteoglycans	21d	4.1 ± 1.7	7.6 ± 2.2* ^p=**0,037**^
(n/25 μm^2^)	35d	9.9 ± 2.8	14.2 ± 1.7* ^p=**0,039**^

Measurements were obtained from samples collected on day 7 (7d), day 21 (21d), and day 35 (35d) after experimental injury. Three samples per animal were collected from the central region of the defect. Values are reported as the mean and standard deviation of each group and were compared by ANOVA and the Tukey post-test . * Significantly different from control (*p* < 0.05).

**Figure 1:**
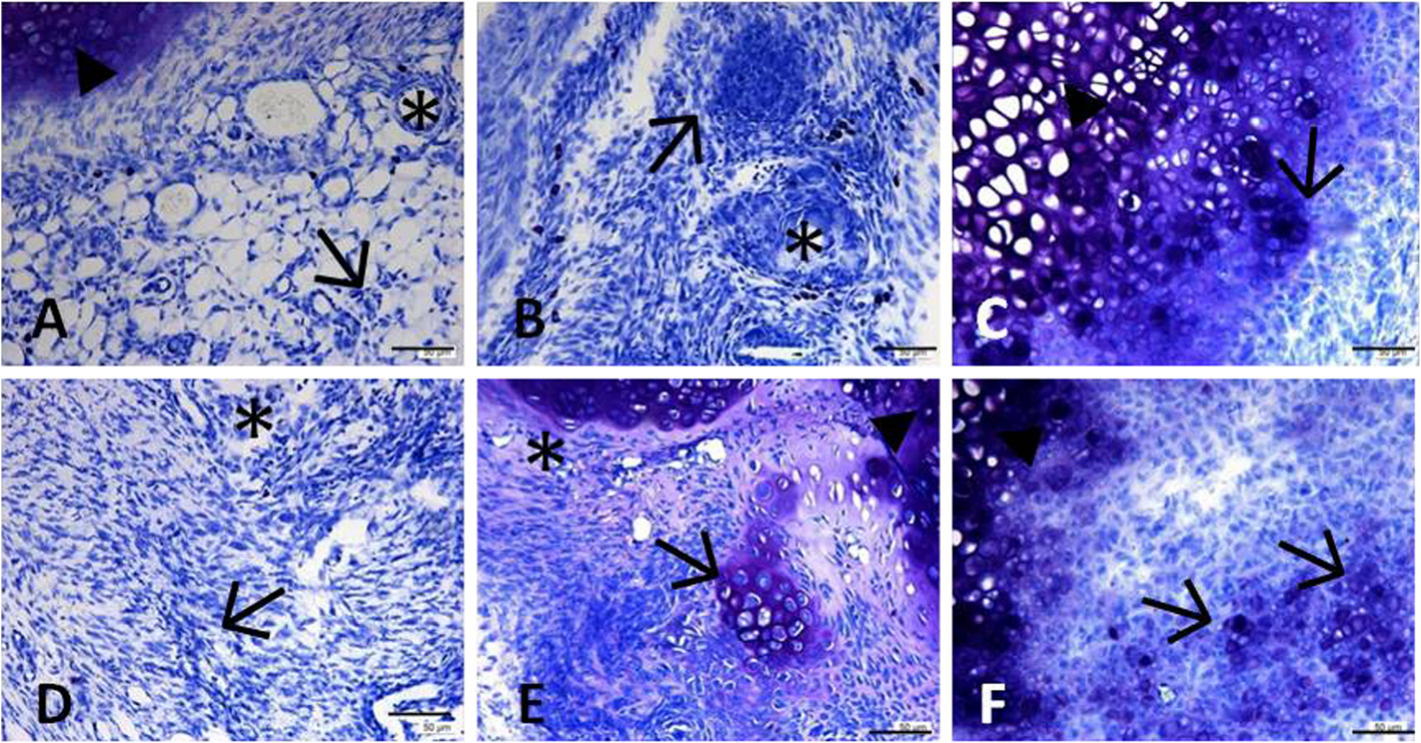
**Photomicrographs of longitudinal sections of the xiphoid cartilage defect area in 45-day-old male rats. A-C**: Control group; **D-F**: group submitted to microcurrent stimulation (20 μA/5 min). Specimens were collected 7 (**A** and **D**), 21 (**B** and **E**), and 35 (**C** and **F**) days after experimental injury. Sections were stained with Toluidine blue in McIlvaine buffer, pH 5. (*) Blood vessels; (→) fibrobastic cells; (▸) original cartilage. Bar, 50 μm.

Toluidine blue staining (Figure 1) revealed a larger number of connective tissue cells in treated animals on day 7 when compared to the control group. In addition, a higher concentration of connective tissue cells organized in central islands was observed in treated animals on day 21. New tissue formation accompanied by intense basophilia at the defect margins was seen in treated animals on day 21 and especially on day 35 when compared to the respective control animals. Only few blood vessels were observed in either group 35 days after experimental injury. Morphometric analysis showed a significant and gradual increase in the total number of connective tissues cells over time in the repair area of treated animals when compared to the respective control group (Table 1). The total number of granulocytes was significantly higher in treated animals on days 7 and 21. However, no difference in the total number of granulocytes was observed between groups 35 days after experimental injury (Table 1).

**Figure 2:**
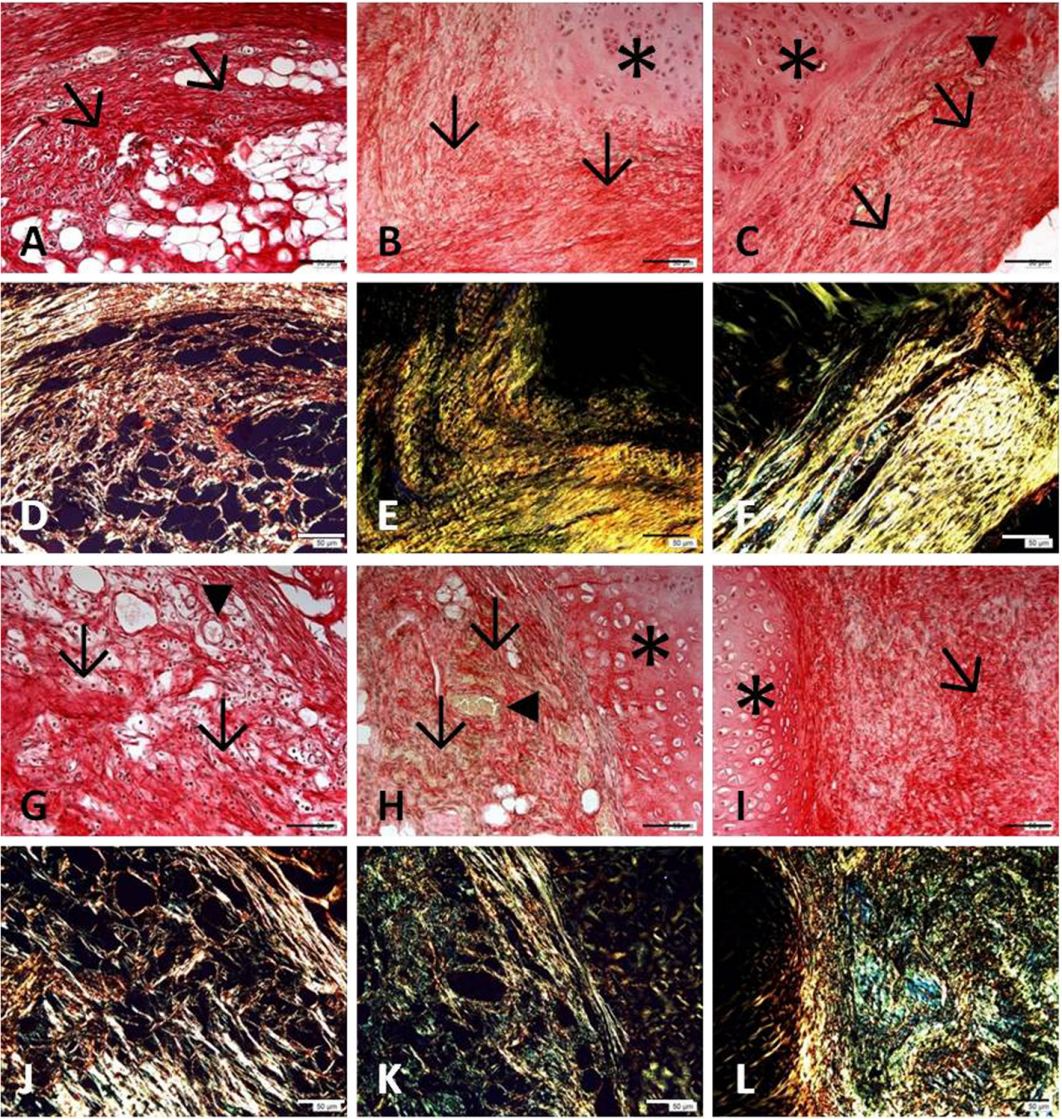
**Photomicrographs of sections stained with picrosirius-hematoxylin and analyzed by bright-field microscopy under polarized light (D-F and J-L). A-F: Control group; G-L: treated group.** Specimens were collected 7 (**A**, **D**, **G**, and **J**), 21 (**B**, **E**, **H**, and **K**) and 35 (**C**, **F**, **I**, and **L**) days after experimental injury. (*) Original cartilage; (→) collagen fibers; (▸) blood vessels. Bar, 50 μm.

Analysis of sections stained with picrosirius-hematoxylin and examined by bright-field microscopy under polarized light (Figure 2) showed a predominance of thick collagen fibers in the control group and thinner fibers in the treated group at all time points studied. A significant difference in the area occupied by birefringent collagen fibers was only observed on day 35, with the percentage being higher in control animals (Table 1). Small numbers of elastic fibers arranged around islands of original cartilage were detected in sections stained by the Verhoeff obtained from the two groups at all time points (data not shown). Alizarin red staining revealed no relevant calcification in the defect area of control or treated animals. However, some calcification points were seen at the defect margins of treated animals on day 35 (data not shown).

**Figure 3:**
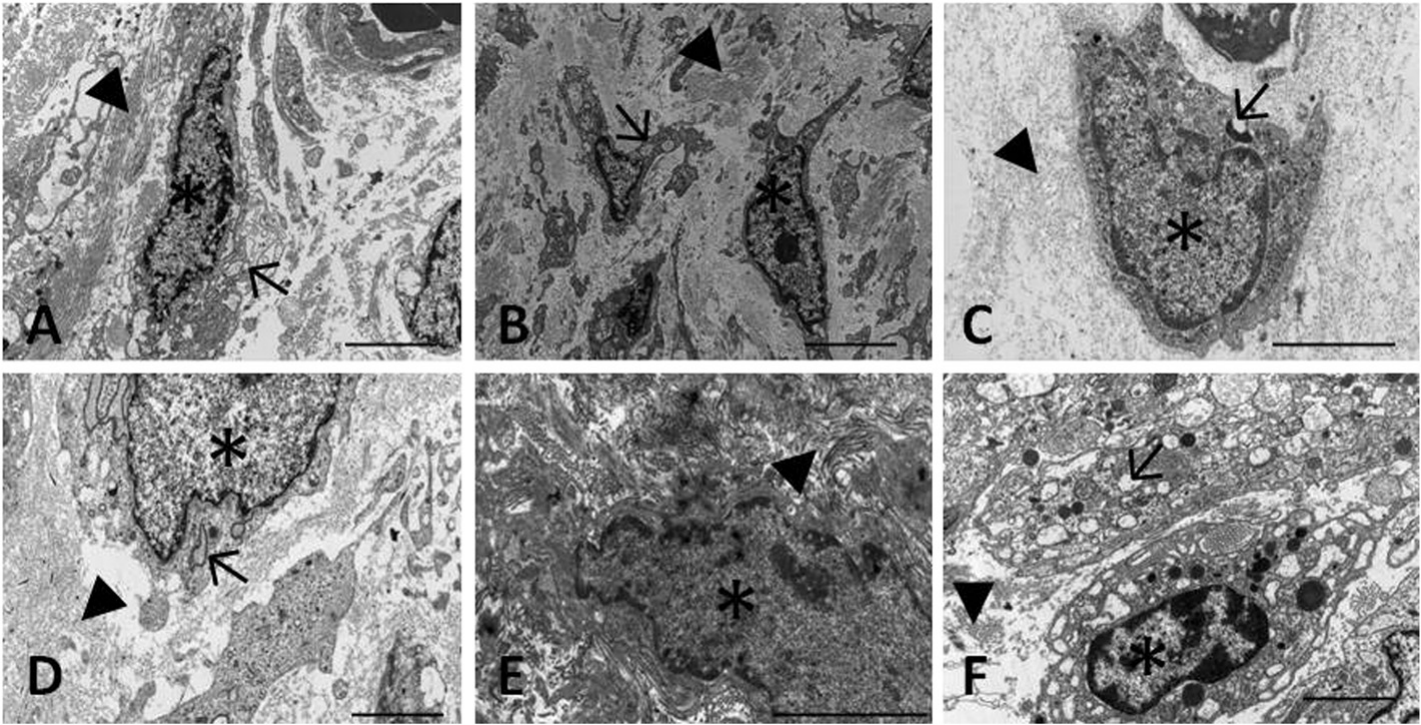
**Electron photomicrographs of the xiphoid cartilage defect area in 45-day-old male rats. A-C**: Control group; **D-F**: group submitted to microcurrent stimulation (20 μA/5 min). Specimens were collected 7 (**A** and **D**), 21 (**B** and **E**), and 35 (**C** and **F**) days after experimental injury. The samples were fixed by the addition of 0.1% tannic acid. (*) Fibroblastic cell; (▸) fibrillar matrix; (→) secretory vesicle. Bar, 2 μm.

Electron photomicrographs of the repair tissue treated by the tannic acid method are shown in Figure 3. Fibroblast-like cells were observed in control animals on days 7 and 21. These cells were surrounded by a fibrillar pericellular matrix that was looser than that seen in the region of the territorial matrix. In contrast, chondroblast-like cells characterized by cytoplasm rich in secretory vesicles and abundant rough endoplasmic reticulum predominated in control animals on day 35. The pericellular matrix consisted of a network of more compacted collagen fibrils than those seen in the territorial matrix. In the treated group, the cells presented chondroblastic features (cytoplasm rich in secretory vesicles) on days 7 and 21. These cells were surrounded by a predominantly fibrillar pericellular matrix. The fibrils had a loose organization. On day 35, chondrocyte-like cells whose cytoplasm contained large amounts of rough endoplasmic reticulum and secretory vesicles were observed in the treated group. The collagen fibrils were arranged in a loose network in the pericellular and territorial region. No difference in collagen fibril diameter was observed between the two groups at the different time points studied (Table 1).

**Figure 4:**
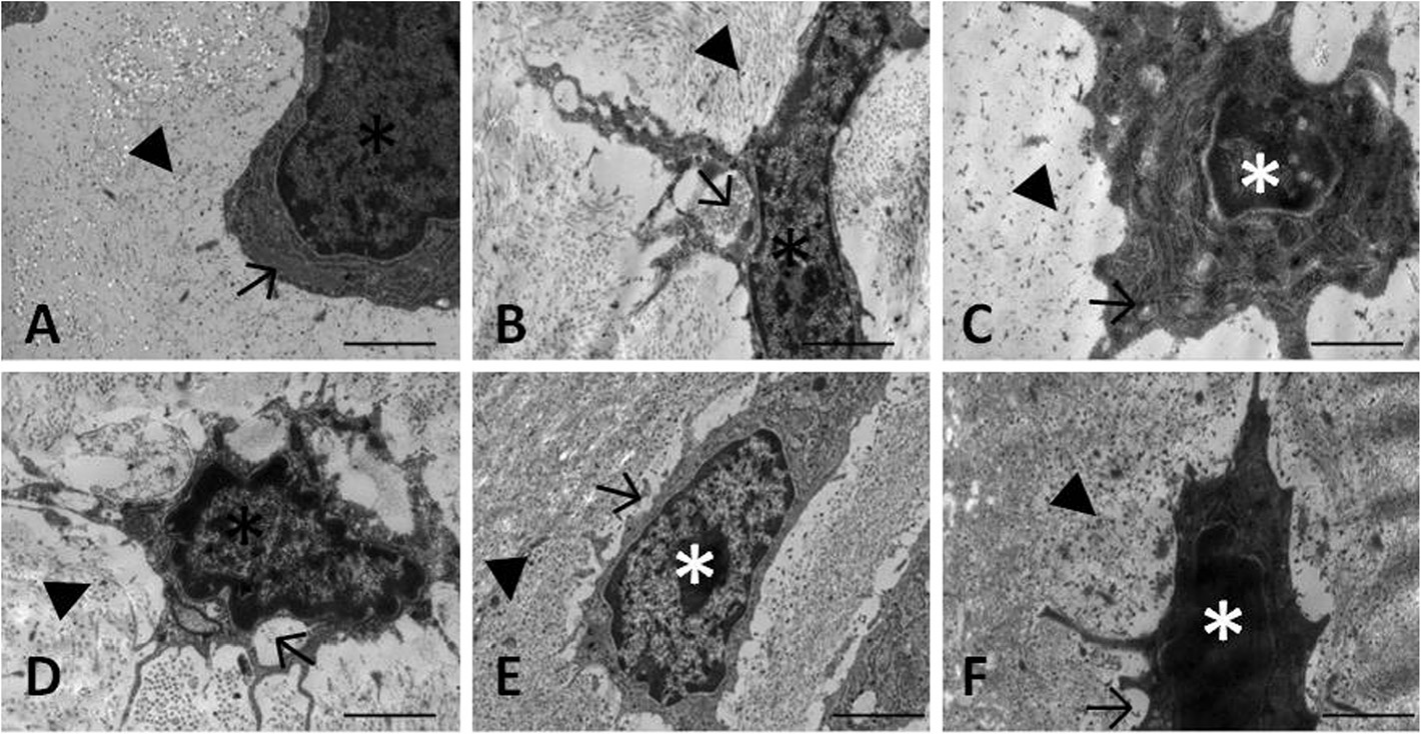
**Electron photomicrographs of sections from control (A-C) and treated (D-F) animals stained with cuprolinic blue for the detection of proteoglycans (PG).** Specimens were collected 7 (**A** and **D**), 21 (**B** and **E**), and 35 (**C** and **F**) days after experimental injury. (*) Fibroblastic cell; (▸) – positive PG staining; (→) secretory vesicle. Bar, 2 μm.

Figure 4 shows electron photomicrographs of the repair tissue submitted to cytochemical staining of PGs. Larger PG complexes were observed in treated animals on day 7 when compared to the control group. In addition, a gradual increase in the size of PGs was observed in treated animals on days 21 and 35 compared to control animals. Treated animals also presented a significantly larger number of positively stained PG deposits on days 21 and 35. The number of stained PGs increased gradually in the two groups over the period studied (Table 1).

## Discussion

Since non-articular cartilage defects and repair are uncommon in clinical practice, studies investigating these processes are important to gain insights into the reorganization of this tissue under different functional conditions of the joints. In a recent study, Moyer et al. [19] observed regeneration of xiphoid cartilage in adult male rats after creating cylindrical defects of different diameters with a dermal biopsy punch. The results were dependent on the size of the defect and on the origin of chondrocytes implanted into the defect. However, the newly formed cartilage contained chondrocytes arranged in columns and abundant PG deposits. According to the authors, a 3-mm cylindrical defect in xiphoid cartilage of male rats aged 7 to 8 weeks is an adequate model to evaluate the potential of non-articular cartilage repair. The same model was used in the present study to analyze the effect of microcurrent stimulation on the dynamics of cartilage repair.

In the present study, only a small amount of newly formed cartilage was observed at the defect margins in all treated animals after 35 days of uninterrupted microcurrent application. These findings agree with the study of Baker et al. [30] who used an *in vivo* model in which a metallic device was implanted into defects created in the femoral condyle of 6-week-old rabbits. Direct electrical stimulation did not lead to complete closure of the defect after 9 weeks of treatment. However, in contrast to the control group that was not implanted with the device, hyaline cartilage was detected in the center of the defect, suggesting that electrical stimulation was able to stimulate cartilage repair.

Another relevant finding was the effect of microcurrent stimulation on the total number of fibroblast cells, which was higher in all treated animals when compared to the respective control group. In addition, the higher concentration of glycosaminoglycans and the earlier organization of cartilage in treated animals may be the result of the beneficial effects of electrical stimulation on the production, maintenance and organization of ECM [6,31]. According to Aaron et al. [32] and Brighton et al. [4], this fact seems to be related to the effect of electrical and electromagnetic field stimulation on the expression of genes encoding ECM proteins, resulting in the increased deposition of cartilage and bone in repair tissue.

Another factor related to the effects of microcurrent stimulation is the induction of the proliferative and differentiation capacity of mesenchymal cells, which are found mainly in young animals [6,32-34]. This capacity increases substantially when the damaged cartilage is surrounded by perichondrium, which is commonly present at sites of non-articular cartilage and was preserved in the animals studied here [35]. The chondrogenic layer of the perichondrium contains a large number of cells with the potential to differentiate. *In vivo* and *in vitro* studies have shown more accelerated repair of cartilage surrounded by perichondrium and that, in contrast to articular cartilage, the newly formed tissue has practically the same properties as the original tissue [9,13].

The abundant presence of granulocytes in treated animals on days 7 and 21 is consistent with one of the most well-known effects of electrotherapy, particularly electrical current stimulation, i.e., the acceleration of the inflammatory process. This phenomenon seems to be related to improvement of the healing and/or repair of connective tissues [36,37]. According to Sonnewend et al. [38], microcurrent stimulation increases ATP production by 500%, with a consequent increase in protein synthesis and tissue regeneration. These authors also demonstrated the beneficial effect of microcurrent stimulation, showing acceleration of the healing process and a reduction of inflammation after 7 days of intervention. However, these benefits were only observed in animals receiving a dosage of 30 μA, whereas in the group receiving a higher dosage (160 μA) treatment was only effective in reducing inflammation at the end of the healing process. This fact agrees with the findings of this study and shows that low-amperage currents reduce the early stages of inflammation.

A larger number of birefringent fibers indicating a larger number of compacted collagen fibers were observed in control animals on day 35. The lack of an effect of electrical current stimulation on fiber compaction might be explained by the fact that low-amperage and low-frequency stimulation only reduces the time of connective tissue healing during the early stages, but has no effect on fiber maturation or reorganization during the repair phase. However, Lee et al. [39] showed that very low doses accelerate skin wound closure in humans. In this case, microcurrent stimulation promoted acceleration of the healing process by increasing collagen fiber deposition, cell proliferation, growth factor concentration, and ATP levels. In addition to these observations, Kirsch and Mercola [40] suggest that electrical sub-sensory stimuli can penetrate cells, restoring the natural bioelectricity after injury. Furthermore, studies have shown that microcurrents can stimulate ion exchange across biomembranes, increasing oxygenation and nutrient absorption by the cells, as well as eliminating catabolites and restoring cell polarity [41-43].

Although the ideal parameters and type of electrical stimulation that is safe and efficient in promoting connective tissue repair, particularly cartilage repair [33,44], have not been established, studies have demonstrated that low frequencies seem to be more efficient in promoting the repair of different connective tissues since they act by altering the membrane potential of the cell [25,32].

In contrast to collagen fibers, the number of elastic fibers seems to be little influenced by microcurrent treatment. Traces of these fibers were observed in the two groups and at the different time points studied. The hyaline nature of xiphoid cartilage does not appear to be influenced by treatment when analyzing the expression of molecules that are generally rare in the stroma of this tissue.

Several studies on cartilage repair have shown that microcurrents tend to induce the formation of fibrocartilage in chondral defects, whereas osteochondral defects are filled with cartilage tissue since the underlying bone promotes vascularization, favoring tissue repair. However, the size of the defects and how they are created will determine the treatment protocol in order to obtain the best results. These facts were described by Lippiello et al. [45] for cartilage tissue. There are no reports investigating microcurrent stimulation of purely articular cartilage defects or even non-articular cartilage defects. Thus, further studies are needed to better understand the processes leading to the calcification points observed at the defect margins in treated animals on day 35 [28,46].

The adequate combination of collagen fibrils and fibers and highly hydrated PGs permits cartilage to withstand the functional requirements during normal activity [4,47]. In the present study, no difference in the mean diameter of collagen fibrils deposited in the repair tissue was observed between the two groups. On the other hand, the number of PGs was increased in treated animals after 21 and 35 days. We believe that during repair the only function of collagen fibrils is to contain the stromal content since the tissue is not submitted to important functional alterations that require the deposition of fibers with variable calibers. However, we did not evaluate the amount of collagen deposited in the tissue during the experimental period to clearly confirm a quantitative effect of microcurrent treatment. On the other hand, the number of stained PGs seems to indicate a positive effect of treatment on the deposition of these molecules in the tissue.

The predominance of chondroblast-like cells in the defect area of treated animals since the beginning of treatment highlights another important aspect of the effect of microcurrent stimulation on the differentiation of mesenchymal cells. Baker et al. [30], Okihana and Shimomura [36], Takei and Akai [48], and Snyder et al. [25], using different electrical stimulation protocols of connective tissue, particularly immature cartilage, demonstrated an increase in the quantity of type II collagen and insoluble PGs, as well as in DNA synthesis and in the morphological differentiation of cellular elements.

## Conclusion

We demonstrated that microcurrent stimulation has shown beneficial effects during non-articular cartilage repair in prepuberal animals. However, the period of observation was not sufficient to evaluate complete closure of the defect. Therefore, further studies will be conducted to evaluate the organization of the newly formed tissue over a longer period of time.

## Competing interests

The authors declare that they have no competing interest.

## Authors’ contributions

CCC, DCZ and LMGN carried out experimental work, data collection and evaluation, literature search and manuscript preparation. JSM helped the surgical procedure and data collection. PPJ helped to prepare and evaluation of electron microscopy analysis. MAME raised funding, supervised research work and refined the manuscript for publication. All authors read and approved the final manuscript for publication.

## Pre-publication history

The pre-publication history for this paper can be accessed here:

http://www.biomedcentral.com/1472-6882/13/17/prepub
